# Fomenting Sickness: Nocebo Priming of Residents about Expected Wind Turbine Health Harms

**DOI:** 10.3389/fpubh.2014.00279

**Published:** 2014-12-12

**Authors:** Simon Chapman, Ketan Joshi, Luke Fry

**Affiliations:** ^1^School of Public Health, University of Sydney, Sydney, NSW, Australia; ^2^Infigen Energy, Sydney, NSW, Australia

**Keywords:** wind farms, wind energy, nocebo, news media, psychogenic health effects

## Abstract

A nocebo effect hypothesis has been proposed to explain variations in where small minorities of exposed residents complain about noise and health effects said to be caused by wind farm turbines. The hypothesis requires that those complaining have been exposed to negative, potentially frightening information about the impact of proposed wind farms on nearby residents, and that this information conditions both expectations about future health impacts or the etiology of current health problems where wind farms are already operational. This hypothesis has been confirmed experimentally under laboratory conditions, but case studies of how this process can operate in local communities are lacking. In this paper, we present a case study of the apparent impact of an anti-wind farm public meeting on the generation of negative news media and the subsequent expression of concerns about anticipated health and noise impacts to a planning authority approval hearing in Victoria, Australia. We present a content analysis of the negative claims disseminated about health and noise in the news media and available on the internet prior to the hearing, and another content analysis of all submissions made to the planning authority by those opposing the development application.

## Background

Australia’s first commercial wind farm commenced operation in 1987, in Western Australia. Over the next 27 years, some 52 farms ranging from small single turbine operations to a 120 turbine 420 MW farm in Victoria became operational (1). Sporadic reports of opposition to some of these wind farms began appearing from about 2002 (2). These objections were initially focused on esthetic, economic, and fauna risk objections to proposed wind farm developments from property owners who believed the turbines would reduce their visual amenity and property values and kill rare or iconic birds and bats. Interest groups with connections to climate change skepticism such as the Australian Environment Foundation (3) also expressed opposition to what they saw as reviled totems of green political values. Several branches of the Landscape Guardians, a movement modeled on the UK’s Country Guardians (4) began to attract minor, sporadic publicity from around 2002.

News reports of a British rural doctor’s claims that wind farms were making people sick (5) saw a Victorian rural doctor make similar claims in 2004 (6) about a 12-turbine wind farm near the town of Toora in Eastern Victoria. He had conducted a small, unpublished survey of 25 residents. At that time, there were 14 operational wind farms around Australia with 135 turbines, none of which had experienced health or noise complaints. In March 2010, the Waubra Foundation was established by a small group of people, none of whom were resident in the community of Waubra in central Victoria. The group, principally through its “medical director,” an unregistered former general practitioner, began publicizing the alleged association between wind turbines and health problems. This included a website, many media interviews and the publication (by a related group) of “symptoms” in a local newspaper (7), listing 12 health problems that residents “should understand could be attributable to wind turbine exposure.”

A 2013 audit of the history of health and noise complaints about wind farms (1) found large historical and geographical variations in wind farm complaints. 33/51 (64.7%) of Australian wind farms had never been subject to noise or health complaints. The entire states of Western Australia and Tasmania had seen no complaints. Just 129 individuals across Australia appear to have ever complained, with 94 (73%) living near six wind farms targeted by anti-wind farm groups (1). The large majority 116/129 (90%) of complainants made their first complaint after 2009 when anti-wind farm groups began to add health concerns to their wider opposition (1). In the preceding years, health or noise complaints were rare despite large and small-turbine wind farms having operated for many years.

The Waubra Foundation, together with several other local cells of opposition centered around a small number of wind farms in South Australia, Victoria, and New South Wales, worked to spread their views about health impacts via local meetings, news media, and the internet. Two Senate enquiries (8, 9) were held in 2011 and 2012, and Australia’s principal independent health and medical agency, the National Health and Medical Research Council, published reviews of the evidence about health impacts in 2010 (10) and 2014 (11). Both reviews concluded there was poor evidence of direct harmful effects. Another 20 reviews published since 2002 have reached broadly similar conclusions about the lack of strong evidence about direct effects (12). Most of these have noted the association of complaints with psycho-social factors, for example, Knopper and Ollson concluded that wind turbine annoyance was “found to be more strongly related to visual impact, attitude to wind turbines and sensitivity to noise” than to distance of complainants’ residences from turbines and that “self reported health effects like feeling tense, stressed, and irritable, were associated with noise annoyance and not to noise itself.” (13).

Given that the variable spatio-temporal distribution of complaints about wind farms in Australia is incompatible with a direct causation theory of noise and health impacts, various psycho-social variables have been noted as being associated with complaining. These include pre-existing negative attitudes to wind farms (13), including views about their impact on landscape esthetics (14), being able to see wind turbines (15), subjective sensitivity to noise (13), having negatively oriented personality traits (16), and deriving economic benefit from wind farms (an apparent complaint-protective factor) (17).

A nocebo effect hypothesis has also been proposed to explain reported complaints about noise and health effects said to be caused by wind turbines (1, 18–23). The hypothesis requires that those complaining have been exposed to negative, potentially worrying information about the impact of wind farms on nearby residents, and that this information conditions both expectations about future health impacts and/or beliefs about the etiology of current health problems where wind farms are already operational. This hypothesis has been confirmed experimentally under laboratory conditions (19, 21), but case studies of the exposure of local communities to such alarming information and any subsequent expressions of concern are lacking.

In this paper, we present a case study of news media and other disseminated negative information and personal testimonies in the months prior to an administrative tribunal hearing to consider objections to the proposed Cherry Tree Range wind farm in rural Victoria, Australia. Australian census data show that the three settlements nearest to the proposed wind farm have the following populations: Trawool (376 dwellings with 789 people), Whitehead’s Creek (159 dwellings with 373 people), and Seymour, 15 km away (2,923 dwellings with 6,370 people). This short video shows views from the planned site for the 16 turbine, 50 MW wind farm^1^).

We present a content analysis of the claims disseminated about health and noise prior to the hearing, and summarize references to future health concerns subsequently made in all public submissions to the Victorian Civil and Administrative Tribunal (VCAT) by those opposing the development application. The developer, Infigen, had taken the issue to VCAT for a decision as the Mitchell Shire Council had not delivered a response about planning permission within the specified timeframe. The Cherry Tree wind farm has not yet been constructed but our data provide baseline information about those publicly expressing beliefs about their concerns about future illness for subsequent potential corroboration of the nocebo hypothesis.

## Methods

### Dissemination of negative information in the local community

Sources of negative information, which might have primed residents to feel concerned about noise and health issues include news media reports and correspondence, a local meeting organized by the wind farm opponents and negative information from the internet, in addition to dissemination of this information through social networks in the area.

All coverage of the proposed wind farm was obtained from a commercial media monitoring company (iSentia) for the period November 9, 2011 through to February 28, 2013. This covered the period from the project’s public announcement until soon after the commencement of the VCAT hearings. Google News was also searched over the same period, using combinations of the search terms (“Cherry Tree”, Cherry Tree, Infigen, “wind farm”). The records retrieved included news, letters, and editorials in local district, state, and national newspapers, but not local radio or statewide television. This material was examined for any negative content about noise and/or health issues under three broad concerns and this data plotted against the total number of items retrieved by the search terms.

The three concerns used to classify items were expressions of concern, or direct assertions:

- that the wind farm would have a direct impact on human health (“Health”)
- that the wind farm would generate audible noise that would cause annoyance or impact on quality of life (“Noise”)
- that the wind farm would have a direct impact on human health through inaudible noise in the infrasonic range (“Infrasound”).

**Figure 1:**
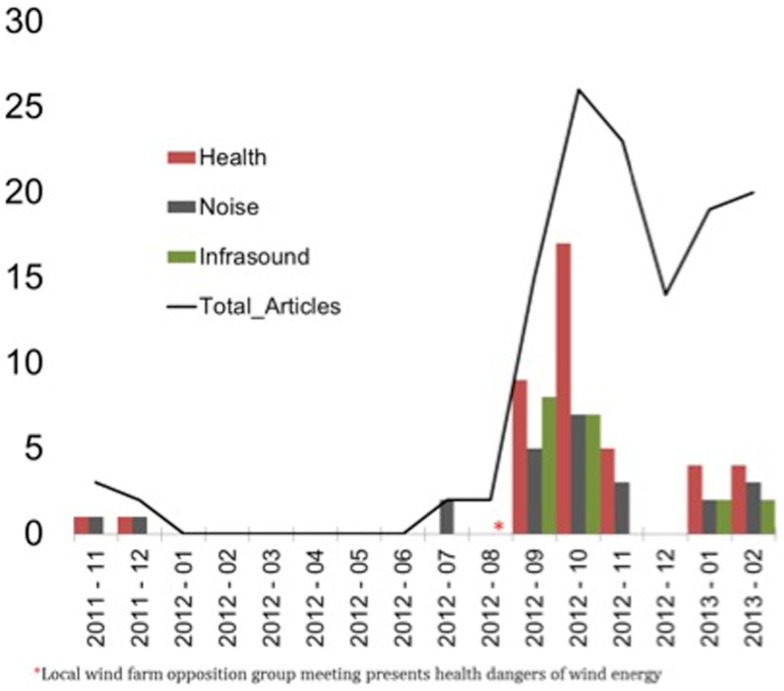
**Incidence of negative news media mentions of health, noise, and infrasound about the proposed Cherry Tree wind farm, Nov 2011–Feb 2013**.

Figure 1 shows the distribution of negative news media coverage. Single new items could be coded for up to three of the above concerns, depending on their content.

On August 28, 2012, a newly proclaimed local anti-wind farm group, the Trawool Valley Landscape Guardians (TWLG), organized a public meeting at Trawool, a small settlement near the proposed site. There was an estimated attendance of 100. The meeting was addressed by two residents (Donald Thomas and Noel Dean) who have property near the Waubra Wind Farm, a road distance of 230 km from Trawool; Max Rheese, a member of the Australian Environment Foundation, an activist group skeptical of global warming and opposed to wind energy; and Steve Campbell, then chief of staff to Senator John Madigan, a minor party politician outspoken in his opposition to wind farms (24).

A video produced by the Waubra Foundation was shown at the meeting^2^, with a director of the Foundation, Kathy Russell, in attendance. Notes taken at the public meeting by an attendee were provided to the authors and two news reports highlighted points made by speakers.

The internet also provides ready access to an abundance of claims about diseases and symptoms said to occur in humans and animals exposed to wind turbines. Some 236 such problems have been cataloged since a collection began in early 2012 (25). The first page of a Google search accounts for 91.7% of all reader click-throughs (26). On August 21 2014, we searched Google.au using five different search strings likely to be used by anyone seeking broad information about wind farms and health. We then weighted the results by the click-through data shown in Ref. (26), and calculated the probabilistic click-through rankings of all top 10 sites returned by the five searches (see Table 2).

### Content analysis of anticipated concerns expressed in submissions

Submissions made to the VCAT are public documents. These were searched for opposing submissions made to the “Cherry Tree Wind Farm Pty Ltd v Mitchell Shire Council” consideration of an application by Infigen Energy to construct and operate a 16 turbine wind farm known as Cherry Tree, near the top of a 550 m ridge, some 12 km from the town on Seymour in Victoria. Seventy five opposing submissions were examined for any mention of health concerns, with other concerns also being recorded (see Table 2). Multiple concerns were recorded separately and postcodes of the writers recorded. We provide illustrative examples of these concerns in Table 1 below.

**Table 1:** **Illustrative examples of negative statements about wind farms and health from news media and public meeting convened by opponents**.

Quote	Source
“After attending the urgent community meeting regarding the Cherry Tree wind farm proposal, we are now more than ever gravely concerned members of the community”	Letter from four residents. Seymour Telegraph, September 12 2012
“The major concern of the audience was health including sleep deprivation, increased blood pressure, heart racing, nosebleeds, and constant headaches derived from the noise, vibration, and infrasound produced from the 160 m turbines”	Seymour Nagambie Advertiser, September 4 2012
“Headaches, wanting to vomit all the time, pains in the chest, blood pressure, can’t sleep, sleeping tablets do nothing for you”	Resident featured in Waubra Foundation video screened at Trawool meeting
“Really bad chest pains in the night, and a lot of blood noses, I’d be asleep and then wake up, and my nose would be bleeding. It’s just pretty scary stuff”	Resident featured in Waubra Foundation video screened at Trawool meeting
“Symptoms have been consistently reported in Australia, up to 10 kilometers from homes. Most symptoms disappear when people leave the area, or when the turbines are switched off”	Waubra Foundation video screened at Trawool meeting
[There’s] “not a single credible research paper in the peer reviewed literature stating that chronic wind turbine noise is harmless to human health but there is now over a dozen peer reviewed papers that say the opposite”	Max Rheese, climate change skeptic, wind farm opponent, at Trawool meeting
“It’s the most bizarre thing. It just sounds so weird but you lay down and you can hear the turbines in your pillow”	Waubra resident Donald Thomas, speaking at Trawool meeting

## Results

### Dissemination of negative information in the local community

Of 126 media articles retrieved, 41 (33%) contained concerns about the health impacts of the proposed wind farm. Ninety five per cent of these were published after the anti-wind farm TWLG meeting in August 2012. Figure 1 shows the number of times each concern was expressed in the collated media content, along with the total number of articles and a marker showing the TWLG meeting.

### Negative information from the internet

Using the 5 different search strings, 27 different sites were retrieved in the top 10 hits thus returned. Of these 8 (30%) were stories or pages, which described negative health impacts of wind farms, and 2 were ranked in the top 10 weighted click-ranked sites. The cumulative weighted click rank score of these negative sites was 134 representing 36.6% of the 366.6 clink rank score for sites that did not give mention or emphasis to negative health issues (see Table 2).

**Table 2:** **Sites returned using five different search strings with Google on wind farms and health, ranked by click volume of Google search position**.

Site	Google page rank on five search strings*					Click rank
	1	2	3	4	5	
https://www.nhmrc.gov.au/your-health/wind-farms-and-human-health	1	1	1	2	–	147.6
http://science.howstuffworks.com/environmental/green-science/wind-turbines-health.htm	4	2	3	1	4	77.7
http://www.abc.net.au/environment/articles/2014/02/03/3935067.htm	–	–	–	–	1	32.5
http://www.smh.com.au/environment/link-between-wind-farms-and-health-problems-rejected-by-research-review-20140224-33cw6.html	3	5	4	5	–	31.7
https://www.nhmrc.gov.au/guidelines/publications/eh53	–	–	2	3	–	29.0
http://en.wikipedia.org/wiki/Environmental_impact_of_wind_power	6	6	10	7	3	26.1
http://www.abc.net.au/news/2014-02-24/no-evidence-that-wind-farms-cause-health-problems-nhmrc/5280790	2	–	–	9	–	20.2
https://ama.com.au/position-statement/wind-farms-and-health-2014	–	3	5	–	–	17.9
http://theconversation.com/au/topics/wind-turbine-syndrome	–	–	–	–	2	17.6
http://oto2.wustl.edu/cochlea/wind.html	8	7	6	6	–	15.4
waubrafoundation.org.au	5	–	–	4	–	14.2
http://www.windturbinesyndrome.com/	–	–	–	–	3	11.4
http://blogs.crikey.com.au/croakey/2014/02/25/wind-farms-no-reliable-evidence-of-health-risks-says-national-health-and-medical-research-council/	–	4	–	–	–	8.1
http://ehp.niehs.nih.gov/122-a20/	–	9	7	–	–	6.1
http://rationalwiki.org/wiki/Wind_Turbine_Syndrome	–	–	–	–	5	6.1
http://ramblingsdc.net/Australia/WindHealth.html	–	10	–	8	–	5.9
http://blogs.crikey.com.au/croakey/2013/09/19/wind-turbine-syndrome-farm-hosts-tell-very-different-story/	–	–	8	10	–	5.5
https://newmatilda.com/2014/01/16/abbott-breathes-new-life-wind-turbine-syndrome	–	–	–	–	6	4.4
http://reneweconomy.com.au/tag/wind-turbine-syndrome	–	–	–	–	7	3.5
http://www.theaustralian.com.au/news/features/what-you-cant-hear-can-hurt-you/story-e6frg6z6-1226252801681?nk=fc182926b58a8a955bdd562e950f16ff	7	–	–	–	–	3.5
http://blogs.telegraph.co.uk/news/jamesdelingpole/100227983/wind-turbines-are-a-human-health-hazard-the-smoking-gun/	–	8	–	–	–	3.1
http://www.abc.net.au/news/2011-03-29/windturbinesicknesspreventedbythedruge2809cmoneye28/45730	–	–	–	–	8	3.1
http://www.cfp.ca/content/59/5/473.full	–	–	9	–	–	2.6
http://www.independentaustralia.net/environment/environment-display/the-ugly-landscape-of-the-guardians,3549	–	–	–	–	9	2.6
http://theconversation.com/study-finds-no-evidence-wind-turbines-make-you-sick-again-23621	10	–	–	–	–	2.4
http://www.abc.net.au/news/2012-05-23/chapman-mass-hysteria/4028112	–	–	–	–	10	2.4

*Search strings: 1, wind farms health problems; 2, wind farms health risks; 3, wind turbines health risks; 4, wind turbines health problems; 5, wind turbine syndrome*.

*Shaded sites are sites with negative content about wind farms and health*.

### Content analysis of anticipated concerns expressed in submissions

There were 75 submissions made from 53 households (some sent separate submissions by different family members). Of the 53, 14 came from Trawool households (representing 3.7% of residences), 16 from Whitehead’s Creek 9.5 km away from the site (10.1% of residences), and 13 from Seymour, 12.7 km away (0.4% of residences). Three were sent from Melbourne addresses (110 km away) and two from known interstate anti-wind farm activists (although interestingly, one used a local address in the area). The remaining five were from hamlets at direct distances ranging from 4.6 to 26.4 km from the proposed wind farm site.

All but one submission mentioned health concerns, with reduced visual amenity and bird deaths also being commonly mentioned. Thirty three (44%) of submissions together named 28 different symptoms or health concerns, with the most common being sleep problems (17 mentions), headache/migraine (11), anxiety (9), stress (8), tinnitus (6), and memory loss, nausea, and hypertension (each five) (Table 3).

**Table 3:** **Concerns expressed in 75 submissions opposing the wind farm development**.

Concerns expressed	*N* (% of submissions)
**Health related**
General concern about health impacts	74 (99)
Concern that sound or noise will cause health impacts	58 (77)
Specific symptoms, illnesses named	33 (44)
Anticipated abandonment of home	17 (23)
More research needed on health impacts	17 (23)
Blade glint/shadow flicker	14 (19)
Concerns pre-existing illness will worsen	11 (15)
Electromagnetic interference	10 (13)
Comparisons with tobacco, asbestos or lead as previously benign re health	4 (5)
**Economic impact**
Visual amenity marred	57 (76)
Fire risk	47 (63)
Traffic and access problems	37 (49)
Loss of tourism	21 (28)
Decline in local business	15 (20)
**Other**
Fauna deaths (esp. birds)	64 (85)
Flora destruction	37 (49)
Community divisiveness	16 (21)
Concern over wind company’s multi-national status	15 (20)
Belief wind farms are uneconomic	12 (16)

Across the 75 opposing submissions, there were many examples of people expressing concern after having been exposed to alarming, negative claims, and testimonies from victims, and scientists and doctors. These were often sourced from the anti-wind farm movement, and particularly the Waubra Foundation, and the TWLG public meeting.

For example:

“*After reading and hearing many accounts of anecdotal evidence given by people living within the vicinity of the wind turbines we are concerned that the turbines may impact on our health. Although we were unable to find any published research on the health problems associated with wind turbines we feel that it better not to take the risk until appropriate research is carried out*.”

“*Innumerable letters and reports have been written by general practitioners who have witnessed first-hand the negative effects of wind turbines on the health of patients in their community. The results are alarming to say the least*.” [from a chiropractor].

Exposure to people claiming to have been made ill by turbine exposure was repeatedly mentioned: “*seeing how sick people have become horrifies us*.”

Many submissions referred to “research conducted by the Waubra Foundation,” despite the organization having recently declared that they do not conduct medical research: “From research from the conducted Waubra Foundation[*sic*] and international acoustic technicians, we know that the following medical conditions have been identified in people living, working, or visiting within 10 km of operating wind turbines.” No submissions showed awareness that those involved with that organization have not conducted any research authorized by any human ethics review committee, nor published any research on the area in an indexed peer reviewed journal.

Wind farm opponents have circulated the factoid that “over 40” Australian families have abandoned their homes (27). “Walking off farms” was mentioned in 17 (23%) of submissions: “*This is evidenced by the fact people are walking off their farms and leaving their houses as a result of the health effects*” and “*I believe at this stage that there is too much evidence of people becoming sick and even having to walk off of their land in other areas because of the negative effects of the turbines*.” Those leaving were said to include turbines hosts “*People who have permitted to have put wind turbines on their property, have had to leave their homes because of illness, problems sleeping and noise*.” We are aware of only one turbine host who claims to have moved because of turbine noise, although serious questions remain about the reliability of claims made by the person concerned (28). One submission threatened abandonment before any adverse effects were experienced: “*I won’t wait to become sick, I would leave*.”

The specter of a distant and venal transnational corporation putting profits over local residents’ health was raised in 20% of submissions. The company concerned, Infigen, operates wind farms in Australia and the USA. Significantly, Australia’s only community-owned wind farm at St Leonards Hill also in Victoria has not been spared minority opposition despite its ownership status. “*One must ask the question of what is more important- that a multi-national corporation generates higher profits or that the mental and potentially physical health of the local community is compromised by allowing the wind farm to operate in this location*.”

Three quarters of submissions expressed concern that existing health problems would be exacerbated: “*My eldest son and mother-in-law, suffer from severe migraine headaches, often brought on by changes in air pressure, always exacerbated by any loud or ongoing noise. The noise from the wind turbines would make their condition unbearable*.”

One submission referred directly to claims made by the two Waubra residents who had addressed the meeting: “*One man got sick and he sold his farm because the wind turbines made him sick. The other man could hear the wind turbine noise in his pillow*.”

## Discussion

Our results describe the dramatic increase in expressed concerns about health and other issues published in local news media immediately following a public meeting organized and addressed by dedicated opponents of wind farms from outside the area. The meeting exposed the small proportion of local residents in attendance to a powerful mixture of sometimes emotional testimony from two complainants from another community, and to contributions from the Waubra Foundation presumably intended to provoke health concerns in those attending and in the social networks with whom they were connected. Our data show that anyone searching the internet in Australia for information on health and wind farms will readily find negative material published by opponents.

Confirmation bias is a well-documented cognitive heuristic where people search for, interpret, and prioritize information in ways that confirm their beliefs (29). If individuals have been primed by exposure to events like the Trawool meeting to understand that wind farms threaten health, subsequent searching for information to incorporate in written submissions may see confirmation bias operate and negative information consonant with those negative beliefs selected.

Victim testimony can be a powerful ingredient in fomenting anxiety in those exposed to their claims. As has been noted in a study of Dutch media coverage “Scientists, technicians, and experts get significantly less space, than laypeople, government, industry, and interest groups, in media coverage of EMF health impacts.” So here too, local news media highlighted this ingredient in its reportage: “*Mr Dean said he suffered balance-related problems which he believed were caused by low frequency sound waves generated turbulence created by wind coming into the turbines. He had suffered head pains, tinnitus and muscle spasms. He had sold most of his land ‘and got the hell out of there*”’ *telling the audience* “*I hope other people don’t have to go through what we’ve gone through*” (30).

An audience member thanked the speakers and said “*I think it’s been extremely informative. A lot of the health issues have come out that we probably weren’t aware of*.” However, one matter that did not “come out” in the Trawool meeting was a detail from a 2012 public submission from Mr. Dean to a Senate enquiry where he stated: “I have been in brain training care and rehabilitation for about 10 years because of an unfortunate, unrelated accident.” (31) and his belief that “The frequencies produced by the turbines are the same as those that operate the brain, the interference of frequencies of the brain by those that are produced by the turbines is why the lower parts of our bodies went cold.” (31).

The Waubra wind farm commenced operation May 2009, so Mr. Dean would appear to have been in rehabilitation for a head injury for some 5–6 years prior to that time and still required this care during the period in which he attributed various adverse health conditions to his exposure to the turbines near his property. He once told another anti-wind farm meeting at Baringhup in Victoria that electricity generated by wind turbines started charging his cell phone without it being plugged in “*I’ve had my* … *mobile phone go into charge mode in the middle of the paddock, away from everywhere*.” (32) This extraordinary claim would certainly be of great interest to manufacturers of mobile phones.

The meeting provided attendees no exposure to the many who live near wind farms who have no noise or health complaints. A selection of such people from the Waubra area can be seen on this video^3^ featuring landowners talking about their experiences of hosting turbines.

The Trawool public meeting provided a concentrated and memorable set of highly negative claims that were followed by a surge in media local reportage, although only one-third of this raised negative issues about wind farms and noise or health. However, in total, objections submitted to VCAT were sent by members of only 53 residences out of 3458 (1.5%) in the Trawool, Whitehead’s Creek and Seymour townships. Australian qualitative research of attitudes toward wind farms in nine eastern Australian wind farm communities found there was “strong community support for the development of wind farms, including support from rural residents who do not seek media attention or political engagement to express their views.” (33).

There was considerable evidence of shared identical wording among submissions. For example, six contained an identical paragraph disputing the wind company’s statement that the noise of wind turbines would be comparable to background noise at a beach. These similarities suggested networking between opponents.

The VCAT decision has since allowed the wind farm to proceed. Of health considerations, VCAT rejected direct causation hypotheses about wind turbines and adverse health outcomes, implying that psychogenic variables were relevant to understanding such experiences:

“The Tribunal has no doubt that some people who live close to a wind turbine experience adverse health effects, including sleep disturbance. The current state of scientific opinion is that there is no causal link of a physiological nature between these effects and the turbine.” and that “The totality of material before the Tribunal suggests, but does not conclusively prove, that these effects are suffered by only a small proportion of the population surrounding a wind farm.” and “The position now, as then, stated by the NHMRC in summary, is that there is no evidence that wind turbines cause adverse health effects.” (34).

Every Australian planning case to date considering the issue of “wind turbine syndrome” has found the evidence offered by proponents of the disease to be insufficient (35). Despite this, health and noise objections persist in the majority of planning cases.

The continued prominence of this issue in wind energy planning cases is linked not to legal utility but more likely to the resonance of received negative information about health impacts of wind farms, and its subsequent repetition through news media and in submissions. This effect is likely to reflect the various “fright factors” that characterize environmental threats with greater propensity to cause outrage in communities. These include factors such as involuntary exposure, perceived inequitable or unfair distribution of risk, “industrial” rather than “natural” risks, untrustworthy sources, and dreaded consequences (36, 37).

An under-explored component of the mechanics of nocebo priming is the attribution of symptoms commonly found in any community to exposure to feared or disliked technology like wind turbines. A recent New Zealand study found that sleep problems, headache, and anxiety, among the most common symptoms mentioned in the Cherry Tree submissions, occur in 28.6, 35.4, and 14.1% of the population, respectively (38). It is possible that some individuals who expressed concerns in their submissions therefore already experience some of the common symptoms highlighted by wind farm opposition groups prior to the development of the Cherry Tree facility. Any future claims that the wind farm, once operational, might be causing these problems would need to be considered against the medical records of such complainants prior to the farm becoming operational.

The VCAT submissions and signed letters to local newspapers by those opposing the Cherry Tree wind farm are all public documents. As such, they provide researchers with data that may be useful in testing the hypothesis that the nocebo priming we have reported translates into symptom reporting after the wind farm commences operation. A future study might select residents living a similar distances to the wind farm who had not attended the “frightening” anti-wind farm meeting and who had no or poor recall of any local media coverage of likely adverse health effects. The symptom profiles of this group could then be compared to those who had made submissions to VCAT as a “real world” test of nocebo priming.

Finally, it is interesting to compare the Cherry Tree Wind Farm development with the Coonooer Bridge Wind Farm, developed and approved at a similar time in Victoria. The latter utilized a community sharing model to distribute income equitably among wind farm neighbors. A scientist from the CSIRO noted in media coverage of the two projects: “When we dug a little deeper, we often found their opposition was based more on concerns about process” (39). Though our research shows the clear impact of the activities of anti-wind farm groups on the expression of health concerns, further research may shed light on what inspires both project resentment and acceptance, which may be highly relevant to the expression of health concerns.

We suggest that research into anti-wind activism comparing varied models of wind energy development will lead to greater knowledge of the dynamics and predictors of health fears around large-scale clean technology projects.

## Conflict of Interest Statement

Simon Chapman provided and was remunerated for expert advice on psychogenic aspects of wind farm health complaints by lawyers acting for Infigen Energy in the Cherry Tree VCAT case described in this paper. Ketan Joshi is employed by Infigen Energy. Luke Fry has no conflicts of interest to declare.

## Footnotes

^1^http://youtu.be/JpxNcTCWXIQ

^2^http://www.youtube.com/watch?v=IEh3sooKU8A&list=UUR4vygYsR-WBQ5y2fX-A7Iw

^3^http://www.vicwind.org.au/waubra-videos
