# Corrigendum: Fomenting Sickness: Nocebo Priming of Residents about Wind Farm Health Harms

**DOI:** 10.3389/fpubh.2015.00234

**Published:** 2015-10-12

**Authors:** Simon Chapman, Ketan Joshi

**Affiliations:** ^1^University of Sydney, Sydney, NSW, Australia; ^2^Infigen Energy, Sydney, NSW, Australia

**Keywords:** wind energy, wind farms, wind farm noise, nocebo, psychogenic illness, news media

In our 2014 paper *Fomenting sickness: nocebo priming of residents about wind farm health harms* (1), a transcription error when typing text from reference #31 saw the word “accident” included in the text of a paragraph in the discussion section of the paper, when the original word was “incident.” The unspecified incident resulted in Mr. Dean being “in brain training care and rehabilitation for about 10 years.” Mr. Dean has stated that the incident was not a head injury and noted correctly that the date of his submission to the Australian Senate committee was 2011, not 2012 as we stated in error. This means that the unspecified incident which lead to some 10 years of “brain training and rehabilitation” should be corrected to read “brain training and rehabilitation for a pre-existing problem” which occurred some 8 years prior to the first year of operation of the Waubra wind farm.

Accordingly, we advise that paragraphs 4 and 5 of the Discussion section should now read:

An audience member thanked the speakers and said “*I think it’s been extremely informative. A lot of the health issues have come out that we probably weren’t aware of*.” However, one matter that did not “come out” in the Trawool meeting was a detail from a 2011 public submission from Mr. Dean to a Senate enquiry where he stated: “I have been in brain training care and rehabilitation for about 10 years because of an unfortunate, unrelated incident” (2) and his belief that “The frequencies produced by the turbines are the same as those that operate the brain, the interference of frequencies of the brain by those that are produced by the turbines is why the lower parts of our bodies went cold (2).

The Waubra wind farm commenced operation May 2009, so Mr. Dean would appear to have been in rehabilitation for a pre-existing problem for some 8 years prior to that time and still required this care during the period in which he attributed various adverse health conditions to his exposure to the turbines near his property. He once told another anti-wind farm meeting at Baringhup in Victoria that electricity generated by wind turbines started charging his cell phone without it being plugged in “*I’ve had my* … *mobile phone go into charge mode in the middle of the paddock, away from everywhere*” (3). This extraordinary claim would certainly be of great interest to manufacturers of mobile phones.

## Conflict of Interest Statement

Simon Chapman provided and was remunerated for expert advice on psychogenic aspects of wind farm health complaints by lawyers acting for Infigen Energy in the Cherry Tree VCAT case described in this paper that is the subject of this corrigendum. Ketan Joshi is employed by Infigen Energy.

## References

[1] Chapman S Joshi K Fry L. Fomenting sickness: nocebo priming of residents about wind farm health harms. Front Public Health (2014) 2:279. 10.3389/fpubh.2014.00279 25566521 PMC4264329

[2] Dean N Submission to the Senate in Regard to Adverse Health Effects of Wind Farms (2011). Available from: https://archive.is/myqAR

[3] Chapman S Wind turbine syndrome: farm hosts tell very different story. Conversation (2013). Available from: https://theconversation.com/wind-turbine-syndrome-farm-hosts-tell-very-different-story-18241

